# Dissociated Accumbens and Hippocampal Structural Abnormalities across Obesity and Alcohol Dependence

**DOI:** 10.1093/ijnp/pyw039

**Published:** 2016-04-22

**Authors:** Tom B. Mole, Elijah Mak, Yee Chien, Valerie Voon

**Affiliations:** Department of Psychiatry, University of Cambridge, Addenbrooke’s Hospital, Cambridge, UK (Dr Mole, Mr Mak, Ms Chien, and Dr Voon); Behavioural and Clinical Neurosciences Institute, University of Cambridge, UK (Dr Voon).

**Keywords:** hippocampus, accumbens, reward

## Abstract

**Background:**

Processing of food and drug rewards involves specific neurocircuitry, and emerging evidence implicates subcortical abnormalities, particularly the nucleus accumbens and hippocampus. We specifically hypothesized that these 2 established regions in addiction neurocircuitry are associated with distinctive in vivo structural abnormalities in obesity and alcohol dependence.

**Methods:**

To specifically investigate anatomically discrete volumetric changes associated with overconsumption of different rewards, we acquired T1 MRI data from 118 subjects in 3 groups comprising obesity (n=42), alcohol dependence (n=32), and healthy volunteer controls (n=44). To exploit novel methods of automated hippocampal subfield segmentation, we used Freesurfer software to generate volumetric data in subject groups for the hippocampal subiculum and its major striatal efferent target, the nucleus accumbens. Hypothesis-led, selective group difference comparisons were analyzed.

**Results:**

We found markedly greater accumbens volumes (*P*=.002) and relatively preserved hippocampal subfield volumes in obesity. Conversely, in alcohol dependence, we found preserved accumbens volumes but atrophy of specific ventral hippocampal subfields, the subiculum and presubiculum. Smaller global subcortical gray-matter volume was found in the alcohol dependence group only.

**Conclusions:**

Reward neurocircuitry including the accumbens and ventral hippocampus may show key structural abnormalities in disorders involving processing of both food and drug rewards, although the foci of disruption may vary as a function of reward modality. Structural differences may subserve altered reward and motivational processes in obesity and alcohol dependence and represent a potential biomarker for therapeutic targeting in key public health disorders.

## Introduction

The relationship between the pathological use of food and drug rewards remains unresolved with evidence arguing for and against overlapping addiction neurobiology (Wang et al., 2004; Avena et al., 2008; Davis and Carter, 2009; Dickson et al., 2011; Ziauddeen et al., 2012). Multiple brain regions are implicated in the processing of drug and nondrug rewards in humans (Koob and Volkow, 2010; Diekhof et al., 2012, 2012; Sescousse et al., 2013). A compelling set of regions is the pathways between the hippocampal subiculum and nucleus accumbens, which have strong anatomical connectivity (Ding, 2013) and are implicated in learning, motivation, memory, and contextual processing affected in addiction (Grace, 2010; Koob and Volkow, 2010).

The hippocampus and nucleus accumbens have a strong anatomical and functional relationship. The accumbens receives major inputs from the hippocampal ventral subiculum (Groenewegen et al., 1987), alongside afferents from the prefrontal cortex and basolateral amygdala (Groenewegen et al., 1999; Haber et al., 2000). These inputs are integrated at the accumbens to initiate motor output and behavior. The accumbens requires hippocampal stimulation for its neurons to depolarize and assume an activated state (O’Donnell and Grace, 1995), and hippocampal activity regulates the spontaneous tonic firing state of dopaminergic neurons through glutamatergic pathways (Grace et al., 2007). Neurotransmission along accumbens-hippocampal circuitry has been implicated in triggering relapse in addiction (Kalivas and Volkow, 2005), highlighting their role as candidate regions that may display underlying structural changes. This is supported by animal models, and a recent study reported that when rodents consumed cocaine, hippocampal stimulation of glutamatergic neurons led to striatal dopamine release and a resumption of drug intake (Vorel et al., 2001). Furthermore, specific hippocampal disruption with reduced dendritic spine density was observed after self-administered morphine. These findings converge to highlight the crucial role of the hippocampal-striatal pathway in disorders of addiction.

In obesity, structural imaging studies have typically reported variable, disseminated, cortical changes. Early structural MRI studies reported nonspecific overall global reductions in gray matter volume in obesity, which correlated with body mass index (BMI) (Ward et al., 2005; Gunstad et al., 2008). Regionally, reductions in brain volume have been shown in several localized areas, although results have been varied. Interestingly, studies have demonstrated negative volumetric correlations with BMI in males, particularly in the medial temporal lobes (Gustafson et al., 2004), anterior lobe of the cerebellum, occipital lobe, frontal lobe, precuneus, and midbrain (Taki et al., 2008). Pannucleus et al. (2006) found lower gray matter areas associated with reward, taste regulation, and behavioral inhibition including the middle frontal gyrus, putamen, and frontal operculum along with reductions in the postcentral gyrus. One study showed higher orbitofrontal-accumbens volume (Horstmann et al., 2011) associated with BMI using voxel-based morphometry, although this has not been confirmed using automated regional segmentation methods. The majority of neuroimaging evidence in obesity has emerged from positron emission tomography (PET) and functional MRI studies (Carnell et al., 2012) implicating neural regions associated with a broad range of processes linked with reward, emotion, memory, cognition, sensorimotor, and taste processing (Carnell et al., 2012). Evidence from human and animal studies has implicated the striatum as having a specific role in obesity (Wang et al., 2001; Guo et al., 2014; Karlsson et al., 2015). Striatal hypoactivity and reduced hedonic value has been proposed as a mechanism of compensatory overeating and increased BMI (Saper et al., 2002). A recent food stimulation PET study reported that higher striatal dopamine release was observed specifically in subjects with comorbid obesity and binge eating disorder compared with obese controls (Wang et al., 2011), further suggesting a specific striatal role in the obesity and binge eating disorder phenotype. Despite the ventral striatum’s highlighted role in modulating eating behaviors (Kringelbach, 2005; Volkow et al., 2008), evidence of structural change is limited.

In alcohol use disorder (AUD), some previous studies have found altered accumbens volumes. In college-aged binge drinkers, we have previously found greater ventral striatal gray matter volumes (Howell et al., 2013). Furthermore, studies investigating chronic alcohol dependence have reported lower accumbens volumes, although methods have differed and the research involved manual tracings (Sullivan et al., 2005) and lateralized regions of interest (ROIs) (Makris et al., 2008) that may contribute to variability in findings. The majority of hippocampal structural imaging studies in alcohol dependence (Agartz et al., 1999; Nagel et al., 2005; Oscar-Berman and Song, 2011; Kühn et al., 2014), but not all (Bleich et al., 2003), have demonstrated hippocampal atrophy. Evidence from animal studies has also indicated lower hippocampal long-term potentiation in response to fear (Stephens et al., 2005). However, assessment of global hippocampal volumes fails to appreciate its rich cytoarchitecture, with well-delineated subfields including the subiculum, presubiculum, and the cornu ammonis (CA). Investigating the differential involvement of these subregions in psychiatric diseases may be important considering previous evidence of histological (Ding, 2013) and functional segregation (Kühn et al., 2014). A recent structural MRI study on 42 patients with alcohol dependence reported localized gray matter neurogenesis and plasticity to the CA 2 + 3 in humans after 2 weeks of abstinence. This study also demonstrated preabstinence volume of these regions negatively correlated with disorder severity, as measured by years of regular alcohol consumption (Kühn et al., 2014), and therefore highlights the importance of hippocampal subfields in disorders involving reward consumption. Using voxel-based morphometry, we previously found structural changes in disorders involving overeating, suggesting potential changes may be amenable to other structural neuroimaging techniques (Voon et al., 2015). Here, we hypothesize that both obesity and AUD are associated with lower ventral striatal and subicular hippocampal subfield volumes in vivo.

## Methods

### Participants

The recruitment, diagnostic criteria, and inclusion and exclusion criteria have been previously reported (Mole et al., 2015; Voon et al., 2015). The study was approved by the University of Cambridge Research Ethics Committee, and all subjects gave full written informed consent. Community-based advertisements were used to recruit subjects over 18 years old in the East Anglia region. Subjects with AUD met DSM-IV-TR criteria (American Psychiatric Association, 2000) for alcohol dependence and were abstinent for 2 weeks to 1 year when scanned. Obese participants had a BMI ≥ 30. To assess for potential effects of subtypes of obesity, obese participants who also met DSM-IV criteria for binge-eating disorder were identified to allow separate subanalyses. A subset of the obese participants (n=22) further met DSM-IV-TR criteria for binge-eating disorder (American Psychiatric Association, 2000). Binge-eating disorder can be defined as a specific obesity phenotype characterized by episodes of eating objectively large amounts of food and feelings of loss of control (Wang et al., 2011). Subjects who smoked tobacco were included in the study. Universal exclusion criteria included current major depressive episode or any history of severe psychiatric disorder (e.g., bipolar affective disorder or schizophrenia) or an active substance use disorder, such as regular cannabis use, assessed using the Mini International Neuropsychiatric Inventory. Subjects were also screened using a drug urine test or breathalyzer on the day of testing and excluded if found to be positive. Subjects further completed The National Adult Reading Test (NART) (Nelson and Willison, 1991). All diagnoses were confirmed by a psychiatrist.

### MRI Acquisition

T1-weighted magnetization-prepared rapid gradient-echo were obtained on a 3.0 Tesla magnetic resonance scanner (Trio, Siemens) with a 32-channel head coil using a tilted plane acquisition, TR=2300ms; TE=2.98ms; FOV 240 x 256 x 176mm; voxel size 1x1x1mm. All images were visually reviewed for exclusion of brain pathology.

### Accumbens and Hippocampal Subfield Segmentation

Subcortical volumes were extracted using a user-independent parcellation process in Freesurfer. Technical details of this protocol have been described previously in detail (Van Leemput et al., 2009). As an overview, nonbrain tissues are removed and a Tailairach transform is applied. Subcortical white matter and deep gray matter structures are parcellated by combining data from voxel intensity, probabilistic atlas locations, and local relationships between anatomical structures. This information is used to automatically assign each voxel to a neuroanatomical label. A Bayesian model based on prior manual tracings is applied.

We used a recently improved pipeline for the automated segmentation of hippocampal subfields (Iglesias et al., 2015). This generates an automated segmentation of the hippocampal subfields based on a novel atlas built primarily upon ultra-high resolution (~0.1mm isotropic) ex vivo MRI data. Technical details for this procedure have been described (Iglesias et al., 2015). As we did not hypothesize laterality effects, left and right ROIs were combined to minimize the number of statistical comparisons. From the outputs of the segmentations, we analysed mean volumetric measures of the nucleus accumbens, cornu ammonis (CA) 2-3, CA4, subiculum and presubiculum.

Group difference effects for the segmented subcortical and hippocampal subfield volumes were assessed using an ANCOVA controlling for age, sex, NART performance, and intracranial volume. To assess for potential effects of smoking status on the hippocampus (Durazzo et al., 2013), smoking status was also controlled for. Results were Bonferroni corrected for multiple comparisons, with *P*<.01 considered significant (0.05/5; 1 accumbens and 4 hippocampal subfield ROIs). Subcortical volumes were examined for partial correlations with BMI and Binge Eating Scale (BES) in SPSS controlling for age and total intracranial volume. We further assessed the relationship between BMI and AUDIT (Alcohol Use Disorders Identification Test, Saunders et al., 1993) with regions identified to be significantly different in the group analysis using partial correlations controlling for intracranial volume and age with *P*<.05 considered significant. On an exploratory level, the relationship between AUDIT and the other subfield regions was assessed.

## Results

### Demographics

Subject characteristics can be found in Table 1. Groups were comparable in age and gender composition and showed no statistically significant differences for these parameters. Obese and AUD groups had significantly higher NART errors and BDI scores than controls (*P*≤.05). Clinical variables were available for BMI (n=103), BES (n=87), AUDIT (n=95, scores unavailable for obesity group), and alcohol abstinence duration (n=25).

**Table 1.** Subject Characteristics

	**Obesity**	**ETOH**	**HV**	**ANOVA F/ *χ*** ^***2***^ **, *P***	**t / *χ*** ^***2***^ **, *P***		
					**Obesity vs HV**	**ETOH vs HV**	**Obesity vs ETOH**
n	42	32	44				
Age	44.59±9.859	41.63±10.87	39.23±12.54	1.74, .181	1.77, .079	0.87, .387	-0.797, .428
Sex Male Female	21M 21F	18M 14F	26M 18F	0.323, .851	1.540, .463	0.061, .804	0.285, .594
BMI	33.02±3.26	23.96±2.57	23.57±2.31	122.00, .00	**12.12, .000**	0.33, .746	**-12.414, .000**
BES	17.76±10.67	8.5±10.01	6.86±7.87	10.774, .00	**4.45, .000**	0.60, .552	**-3.288, .002**
AUDIT	6.26±5.86	20.63±12.89	5.08±3.763	30.31, .00	-0.882, .381	**-5.682, .000**	**5.112, .000**
NART errors	15.44±6.789	17.65±7.975	11.98±5.284	4.438, .015	**2.532, .013**	**-2.882, .008**	-1.117, .269
BDI	11.73±8.21	12.92±10.14	6.236±6.92	7.86, .001	**3.07, .003**	**3.19, .003**	0.747, .458

Abbreviations: AUDIT, Alcohol Use Disorders Identification Test; BDI, Beck Depression Inventory; BES, binge eating scale; BMI, body mass index; NART, National Adult Reading Test.

Categorical variables were compared using χ^2^ tests. Continuous variables were examined using *t* tests. Mean (± SD) shown for continuous variables.

### Accumbens and Hippocampal Subfield Volumes

Table 2 lists group regional differences for regional structures. A significant group effect was found in segmented accumbens volumes. Increased accumbens volumes were found in obese participants compared with healthy controls (Table 1). When obesity subgroups were analyzed separately according to the presence or absence of comorbid binge-eating disorder, there were no group differences (*P*>.05). AUD showed lower whole-hippocampal volume findings. Within hippocampal subfields, the subiculum and presubiculum showed the most significantly different volume (*P*<.025). Compared with controls, total subcortical gray matter was lower in the AUD group but not the obesity group.

**Table 2.** Cross-Diagnostic Volumetric Comparisons

**Region**		**Obesity vs HV**		**ETOH vs HV**		**AUDIT Correlations**	
			***P***		***P***	**Corr**	***P***
Ventral striatum	Nucleus accumbens	**↑**	.002		.735	-0.080	.489
Hippocampal subfields	Subiculum		.859	↓	.025	-0.201	.090
	Presubiculum		.801	↓	.008	-0.162	.173
	CA 2 3		.9634		.621	-0.158	.185
	CA 4 DG		.469		.160	-0.255	.030
Whole-hippocampus			.712	↓	.042	-0.306	.007
Global measures			.150	↓	.042	-0.192	.095

Abbreviations: AUDIT, Alcohol Use Disorders Identification Test; ETOH, alcohol dependence; HV, healthy volunteer; NART, National Adult Reading Test.

Comparisons based on ANCOVA. Regions represent bilateral volumes. AUDIT correlations represent partial correlations controlling for age, total intracranial volume, sex, and NART performance and were based on subjects from all groups.

**Figure 1.**
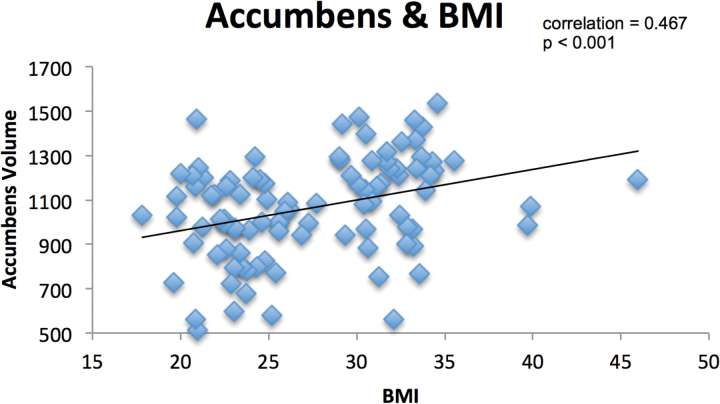
Relationship between accumbens volume and body mass index (BMI). Partial correlation controlling for age, sex, National Adult Reading Test scores, and estimated total intracranial volume.

**Figure 2.**
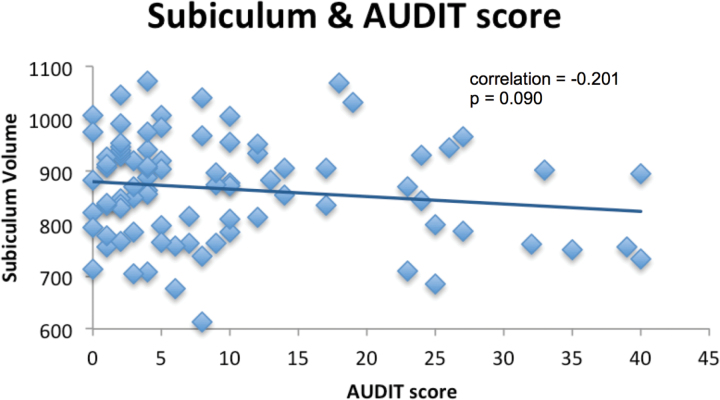
Relationship between subiculum volume and alcohol use disorders identification test (AUDIT). Partial correlation controlling for age, sex, National Adult Reading Test scores, and estimated total intracranial volume.

Correlation analyses revealed that BMI was positively correlated with accumbens volumes across all subjects (r = 0.467, *P*≤.01) (Figure 1), but this BMI correlation was not significant in the obesity group (r = 0.088, *P*=.590) or healthy volunteers. AUDIT scores significantly negatively correlated with volumes of the whole hippocampus volume and CA4 and reached trend level for the subiculum (*P*=.090) (Figure 2). No AUDIT correlations reached significance when either subject group was analyzed separately. No statistically significant correlations were found with the Binge Eating Scale or duration of alcohol abstinence with any region in any population group.

As depression scores were significantly higher in both the obesity and AUD groups, subanalyses with group difference and correlation analyses were conducted with BDI scores as a covariate of no interest. No significant effect of BDI scores on results was found when this was performed, indicating observed effects were independent of depressive comorbidity. Similarly, when smoking status was also controlled for, no significant change in results was observed.

## Discussion

### Main Findings

We show a dissociation in 2 key neural regions implicated in reward neurocircuitry in obesity and AUD. Specifically, relative to controls, in obese subjects we found higher ventral striatal volume, whereas in AUD subjects we found a lower total hippocampal volume, including the presubiculum and subiculum as well as reduced subcortical gray matter. These findings implicate abnormalities in the hippocampal-ventral subiculum neurocircuitry, which involve critical regions shown to be influenced by drugs of abuse (Grace, 2010).

### Accumbens

Previous obesity imaging studies (Taki et al., 2013; Ward et al., 2005; Gunstad et al., 2008) showed lower gray matter volume averaging both cortical and subcortical areas. In the current study, although no total gray matter volume reduction was found, we found increased volume in the accumbens. This regionally specific finding is consistent with a previous finding of accumbens size positively correlating with BMI in a study using subjects with a broad range of BMIs (18–44kg/m^2^), including the healthy range (Horstmann et al., 2011). In contrast, our methods differ from the voxel-based morphometry approach previously applied, using instead an automated algorithm for segmentation of subcortical regions. The study also found BMI correlated with the orbitofrontal cortex and the hypothalamus, consistent with altered coding of reward salience, preference, and central homeostatic signals. Other studies have shown more widespread changes in gray matter in emotion-processing areas (anterior cingulate), taste-processing (frontal operculum, post-central gyrus), feeding-related decisions, and response inhibition (middle frontal gyrus) (Pannacciulli et al., 2006) as well as other areas (anterior cerebellar lobe, occipital lobe, thalamus [pulvinar] and midbrain) (Taki et al., 2008).

In obesity specifically, multiple mechanisms have been proposed to explain distributed reductions in gray matter. Neuronal loss related to age has been shown to be accelerated with disorders that increase ischaemia (Taki et al., 2008), and obesity has been associated with both elevated ischaemia and multiple vascular disorders such as carotid artery wall thickening (De Michele et al., 2002), arterial stiffness (Yki-Järvinen and Westerbacka, 2000), and coronary endothelial dysfunction (Williams et al., 2002). Additionally, brain changes may be mediated by reduced exercise (Colcombe et al., 2003) and impaired respiratory function (Guo et al., 2006). Obesity may also be associated with hypercortisolemia (Björntorp, 2001), which is associated with lower brain volume (Simmons et al., 2000). Altered inflammation is implicated in both obesity (Van Dijk et al., 2005; Raji et al., 2010) as well as alcohol dependence. In contrast to our hypothesis, we observed an increase in ventral striatal volume in obesity. Increases in gray matter volume may be related to multiple underlying cellular and molecular mechanisms. Candidate plasticity mechanisms include use-dependent plasticity, axon sprouting, dendritic branching, synaptogenesis, alterations in glial number or morphology, and angiogenesis (Zatorre et al., 2012). As with other voxel-based morphometry studies, the relative contribution of such mechanisms is ambiguous (Mole et al., 2014), and further study is required to fully explain these differences (Zatorre et al., 2012).

Excessive stimulation of the hippocampal-ventral striatal pathway may lead to adaptive changes in the ventral striatum. In a recent smoking study, using automated subcortical parcellation methods, similar to those used here, left accumbens volume was shown to correlate with lifetime use of cigarettes, consistent with a hypothesis of plasticity effects (Das et al., 2012). However, the correlation was in the opposite direction, where greater left accumbens volumes were associated with lower rather than higher cigarette use. Other studies have not consistently found striatal volume differences in smoking (Brody et al., 2004; Gallinat et al., 2006). We did not detect any effect of smoking status with accumbens volume in the current study. We note that we did not see any differences between obese subjects with and without BED, which may be related to limitations in sample size. A previous study applying voxel-based morphometry methods to the same obese participants as the current study showed that obesity with BED was associated with a lower medial orbitofrontal cortex and ventral striatal volumes relative to those without BED (Voon et al., 2015). Here, obese subjects are compared with healthy volunteers but using a measure of cortical thickness rather than voxel-based morphometry.

### Hippocampal Subfields

Statistically significant reductions in whole-hippocampal or subfield volumes were not found in obesity. The exact reasons for this are unclear. An effect of obesity on hippocampal volumes could potentially be expected given that longitudinal structural studies have identified obesity as an independent risk factor for temporal lobe atrophy cognitive decline and suggest a role in regulating energy intake (Davidson et al., 2007; Gustafson et al., 2004; Taki et al., 2013). It has been shown that obesity may be associated with accelerated nonlinear rates of medial temporal lobe and whole-hippocampal atrophy associated with increased BMI (Gustafson et al., 2004; Taki et al., 2013). Conversely, another large study (n=471) found hippocampal volumes to be enlarged in obesity (Widya et al., 2011). The lack of a difference in the current study could be related to several factors, including sample size or that the current sample focuses on mild to moderate obesity rather than more severe forms of obesity.

In alcohol use disorders, we confirm previous observations of lower whole-hippocampal findings (Agartz et al., 1999; Laakso et al., 2000; Nagel et al., 2005; Hoefer et al., 2014; Kühn et al., 2014) and extend these findings with automated segmentation showing that atrophy appears to significantly affect the subiculum, based on both group differences and clinical correlations. The adjoining presubiculum further showed involvement and is relatively poorly understood in terms of its anatomical and functional characteristics. The presubiculum receives afferents from the medial thalamus, which is thought to be critically involved in alcohol dependence. Regional medial thalamic volume has been strongly linked to severe alcoholism associated with Korsakoff’s syndrome (Pitel et al., 2012). Indeed, thalamic disruption has been described as a cardinal feature of alcoholism (Pitel et al., 2014). The presubiculum has efferents to the medial enterohinal cortex, mammillary bodies, and lateral-dorsal thalamus (van Groen and Wyss, 1990). Unlike the subiculum, the presubiculum does not project to the ventral striatum. Whether the observed presubicular differences are entirely confined to its subfield or represent overlapping structural differences in the adjoining subiculum is unclear. Constraints of MRI resolution and subfield segmentation (Sapolsky, 2000) raise the possibility that the observed differential presubiculum structure may represent regions within the subiculum, as subicular/presubicular boundaries cannot be delineated with histological certainty in vivo. The ventral subiculum has an important role as a context-dependent regulator of dopamine neuron responsivity that responds to environmental exposures and that may be implicated in relapse in alcoholism. The relatively strong negative correlation of AUDIT scores with subicular volume may hence provide a pathological basis that contributes to the high rates of relapse seen in detoxified patients.

In cocaine use, the subiculum has been implicated in reward sensitisation and hyperactivity of the dopamine system. In rodents with inactivated subiculum, the expected sensitization after cocaine use is reversed, and behavioral activation response magnitudes are normalized to levels seen in normal controls (Lodge and Grace, 2008). On exploratory analyses beyond the subiculum, the CA4 subfield was also negatively correlated with AUDIT score. Volume changes may be directly due to ethanol toxicity or indirectly from associated nutritional deficiencies, both interacting with genetic vulnerabilities and environmental influences (Oscar-Berman and Song, 2011). Specifically, chronic alcohol exposure upregulates inflammatory mediators that activate glial cells and promote intracellular signalling pathways involving cyclooxygenase-2, cytokines, and inducible nitric oxide synthase associated with cellular death (Pascual et al., 2007; Guerri and Pascual, 2010). In addition, excitotoxicity and neural injury has been associated with COX-2 and iNOS (Pascual et al., 2007; Guerri and Pascual, 2010).

In this study, we applied the recently updated Freesurfer hippocampal subfield algorithm (Iglesias et al., 2015). While the previous version (Van Leemput et al., 2009) has been widely utilized to investigate subfield involvements across multiple conditions, such as alcohol dependence (Kühn et al., 2014), smoking (Durazzo et al., 2013), dementia (Mak et al., 2015), and Parkinson’s Disease (Pereira et al., 2013), there were several important limitations that affected the agreement of subfield measurements with those of histological studies (Iglesias et al., 2015). By using high-resolution ex vivo images to build the atlas, the reliability of the annotations made by human labellers are improved. Furthermore, by including the “molecular layer” (stratum radiatum, lacunosum molecuare, hippocampal sulcus, and molecular layer of the dentate gyrus), the atlas was able to delineate the internal structure of the hippocampus with more accuracy. As a result, the subfield volumes derived from the new atlas are more compatible with data from histological studies (Iglesias et al., 2015).

The current study used cortical thickness measures, which may be superior to previously used voxel-based morphometry approaches. Cortical thickness, surface-based methods are likely to be more reliable and provide improved registration compared with voxel-based morphometry (Hutton et al., 2009; Clarkson et al., 2011).

This study is not without limitations. Whether the observed structural differences represent vulnerability to obesity and alcohol use disorders, or whether they are a result of exposure cannot be inferred from a cross-sectional design. These findings warrant further longitudinal research to clarify the spatiotemporal progression of structural changes in obesity and AUD. It is also worth considering that although age was used as a covariate, it is possible that this factor influenced results. There is also a continuing debate over the use of BMI as an estimate of obesity, as it does not distinguish fat mass from lean mass or accurately measure intra-abdominal obesity. Its widespread use, however, makes it a clinically relevant and important obesity metric facilitating the generalizability and comparison of findings.

In conclusion, the neural circuitry underlying reward, memory, and learning processes involving the hippocampus and nucleus accumbens appears critically but differentially disrupted in disorders of obesity and AUD. We show that even milder forms of obesity (mean BMI 33) appear to be associated with an increase in ventral striatal volumes. This data is in line with literature suggesting reward sensitivity may be highest in mild obesity and represent an inverted u-shape with the severity of obesity (Davis and Fox, 2008). Moreover, recent evidence suggests the severity of human obesity may also demonstrate a nonlinear relationship with dopaminergic tone (Horstmann et al., 2015). These findings may help explain the previous heterogeneity found in studies of obesity.

In contrast, we show lower hippocampal volumes and particularly presubicular volumes in AUD. Whether these findings represent a premorbid trait or a secondary compensatory phenomenon remains to be investigated, ideally with further longitudinal research. These findings highlight differences between these 2 disorders but also suggest a highly relevant common neural pathway, which may explain common abnormalities in reward processing and motivation. Such neural regions may represent possible future therapeutic targets.

## Statement of Interest

None.
